# Peste des petits ruminants in Northern Sudan

**DOI:** 10.5455/javar.2025.l900

**Published:** 2025-04-16

**Authors:** Intisar Kamil Saeed, Yahia Hassan Ali, Muaz Magzob Abdellatif, Husham Mohammed Ataalfadeel, Anwar A. Alsharari, Ahmed Mohammed Abdel-Mageed, Medhat Ahmed Abu-Tahon, Hind Abdelmajeed Rikabi, Alaa Ahmed Mustafa

**Affiliations:** 1 Department of Biology, College of Science and Arts, Rafha, Northern Border University, Arar, Saudi Arabia; 2 Virology Department, Central Veterinary Research Laboratory, Khartoum, Sudan; 3 Department of Biological Sciences, College of Science, Arar, Northern Border University, Arar, Saudi Arabia; 4 Department of Mathematics, College of Science, Arar, Northern Border University, Arar, Saudi Arabia; 5 National Center for the Prevention and Control of Plants Pests and Animal Diseases, Saudi Arabia; 6 Department of Pharmacy Practice, College of Pharmacy, Rafha, Northern Border University, Arar, Saudi Arabia

**Keywords:** Northern Sudan, PPR, antibodies, antigen, genome

## Abstract

**Objective:**

This work is to elucidate the prevalence of Peste des Petits (PPR) in Northern Sudan through antibody, antigen, and genome detection.

**Materials and Methods:**

Serum and tissue samples from lungs showing pneumonic lesions of goats and sheep were collected in slaughterhouses in both states in Northern Sudan. Sera were examined for antibodies to the PPR virus by Competitive ELISA; Immunocapture ELISA was used for testing PPRV antigen in lung tissues. Some of the ELISA-positive tissues were examined for the PPRV genome by reverse transcription PCR (RT/PCR).

**Results:**

The overall seroprevalence was 35%, with 40.8% in sheep and 28.4% in goats out of 945 sera that were tested. In the River Nile State, the prevalence was 30.1% (34.4% in sheep, and 24.6% in goats). In Northern State, it was 41.8% (50.5% in sheep, 33% in goats). Using IcELISA, the overall PPRV antigen detected in these two states of Northern Sudan was 7.7%; in River Nile State, it was 5% in sheep. In Northern State, 15.4% of sheep and 20% of goat lung tissue samples tested positive. PPR genome could be detected by RT/PCR.

**Conclusion:**

The results pointed to the increased prevalence of the disease in Northern Sudan, especially the Northern State.

## Introduction

Peste Des Petits Ruminants (PPRs) is the most serious infectious disease that affects sheep and goats. The disease results in fever, diarrhea, pneumonia, and respiratory and digestive tract inflammation with up to 90% mortalities [1]. The aetiologic agent is PPR virus (PPRV), a member of the Morbillivirus genus, Paramyxoviridae family [2]. PPR is widely distributed worldwide, in Africa, especially in countries bordering Sudan and other Eastern African countries. In Ethiopia, positive results were seen in 38% of sheep and 69% of tested goat sera [3]. In Kenya, PPRV seropositivity was detected in 51% of goat sera [4]. In South Sudan, PPR cases were confirmed in sheep and goats [5]. PPR seroprevalence and/or antigen was detected in Uganda [6], Egypt [7], Tanzania [8,9], Western Africa, Sierra Leone [10], and North Africa, in Algeria and Morocco [11].

In Sudan, the disease was first nominated in South Gadarif in eastern Sudan in 1971 [12], then in central Sudan during 1971–1972 [12]. PPRV was also isolated in Darfur State [13], the El Hilalia area in central Sudan [14], and in various parts of Khartoum State [15]. PPRV antigen was detected in lymph nodes and ocular and nasal discharge samples of goats [16]. PPRV antibodies were detected in different species in Darfur in Western Sudan [17] and different parts of Sudan [18,19]. The first documented PPR antigen detection in River Nile State at Northern Sudan was reported in 2002 [20]. In 2009, 34% of sheep and goat sera at River Nile State tested positive for PPR antibodies using cELISA [21].

The PPR prevalence in different regions of Sudan was extensively studied; however, its distribution in Northern Sudan was poorly investigated. The first report for PPR seropositivity in Northern Sudan revealed antibody detection in 29% of sheep sera in the northern part, as well as 33% of sheep and 31% of goat sera in the middle part of River Nile State [22]. Then in Northern State, PPR seropositivity was detected in 26% of sheep and 19% of goat sera [23]. PPR seroprevalence in 26% of tested sheep and 40% of tested goats in River Nile State was reported [24]. In 2008, 46% seropositivity was detected in sheep sera from River Nile State [25]. Between 2008 and 2008 and 2008–2012, 63% of sheep and 33% of goat sera were tested positive in the River Nile and Northern States [26]. During 2016–2017, seroprevalence of PPR was detected in 89% of sheep and 100% of goat sera from River Nile State [27]; recently, during 2022, 32% PPR seroprevalence within goats and sheep in Northern State was detected [28]. It was noticed that reports about the prevalence of PPR in Northern Sudan were scarce for the River Nile State and rare for the Northern State. This work focuses on this area to explore the situation of the disease there to aid in its control measures.

## Materials and Methods

### Ethical clearance

The Central Veterinary Research Laboratory has approved this research project. It is an abattoir-based study; samples were collected during slaughtering at slaughterhouses; no direct contact with live animals was adopted.

### Area of the study

This study focused on Northern Sudan, which includes two states, Northern and River Nile.

States.

### Sample size estimation

The formula published by [29] was used to estimate the sample size, with an intended absolute precision of 5%, a 95% confidence interval, and a prevalence of 29%, as reported by [22]. Where *p* is the expected prevalence, d is the target precision, and n is the needed sample size. Upon replacing each value, *n =* 315 is obtained. The sample size increased threefold (*n* = 945) to enhance precision.

$n=\;\frac{1.96^{2}p(1–p)}{d^{2}}$

### Serum sample

Serum samples were collected during slaughtering in slaughterhouses at different localities in the two states in Northern Sudan. There was no history of previous PPR vaccinations in areas of sample collection. In total, 305 sera of sheep were collected from River Nile State (Atbara *n =* 120, Shendi *n =* 60, Barbar *n =* 25, Abuhamad *n =* 100). Goat samples were 240 (Atbara *n =* 70, Shendi *n =* 30, Barbar *n =* 40, Abuhamad *n =* 100). In the Northern State, 200 sheep and 200 goat sera were collected (Dongola, *n* = 100, Marawi, n = 100, for each species). Sera were kept, stored, and dispatched to the Central Veterinary Research Laboratory, Khartoum, to be examined for the existence of PPR antibodies.

### Tissue samples

Tissues were collected from lungs showing pneumonic lesions during slaughtering in slaughterhouses from sheep in the River Nile (*n =* 60) and Northern State (*n =* 13). Only 5 goat samples were collected from the Northern State.

### PPR antibody detection

Using cELISA kits, sera (*n =* 945) were examined for PPR antibodies; kits were kindly provided by Genevieve Libeau of CIRAD (Montpellier, France). The kit was used as instructed.

### Statistical analysis

Processing and analysis of data were done with version 27, SPSS (Statistical Package for Social Sciences). The Chisquare test was adopted to inquire into the correlation between the prevalence of PPR, species, state, and district. Significance was estimated at *p* < 0.01, with a 95% confidence level.

### Detection of PPR antigen

Lung tissues (*n =* 78) were investigated for PPR antigen by immunocapture-ELISA (IcELISA), the kits kindly supplied by Genevieve Libeau of CIRAD (Montpellier, France). The test was carried out following the provided procedure.

### Extraction of RNA

The ribonucleic acid was purified from lung tissues (*n =* 6) and positive control by using Qiagen RNeasy kits, according to the instructions of the manufacturer.

### Reverse transcription PCR (RT/PCR)

Tissues (*n =* 6) were subject to nucleoprotein gene amplification (~352 bp) using NP3 and NP4 primers described by [30], via Qiagen One-Step RT-PCR. In a 45 μl reaction mix, 5 μl of RNA was amplified. The reaction mix contained 10 μl of 5X Qiagen buffer, 2 μl of dNTP mix, 10 μl of Q-solution, and 2 μl of One-Step RT-PCR mix, a final concentration of 0.6 μM for each primer and water. The following thermal condition was set: 30 min of reverse transcription at 50°C, 15 min of initial PCR denaturation at 95°C, and 40 amplification cycles, including 30 sec at 94°C, 30 sec at 60°C, 60 sec at 72°C, and a final elongation for 10 min at 72°C. 10 μl of the products were put in 1.5% agarose gel electrophoresis for documentation.

## Results

### Determination of PPR antibodies

By cELISA, antibodies to PPRV were found in goats and sheep sera in both states. The overall detected seropositivity was 35% (40.8% in sheep, 28.4% in goats); it was 32.8% in River Nile State, 32.2% in sheep, and 33.6% in goats, while it was 46.7% in sheep and 36.3% in goats; details are presented in Table 1.

**Table 1.** Seroprevalence of PPR antibodies among sheep, goats according to location as detected by c-ELISA.

Species		Atbara	Shendi	Barbar	Abuhamad	Dongola	Marawi	Total
Sheep	Count	40	19	7	39	42	59	206
	%	33.3%	31.7%	28.0%	39.0%	42.0%	59.0%	40.8%
Total		120	60	25	100	100	100	505
Goats	Count	25	20	2	12	36	30	125
	%	35.7%	66.7%	5.0%	12.0%	36.0%	30.0%	28.4%
Total		70	30	40	100	100	100	440

**Figure 1.**
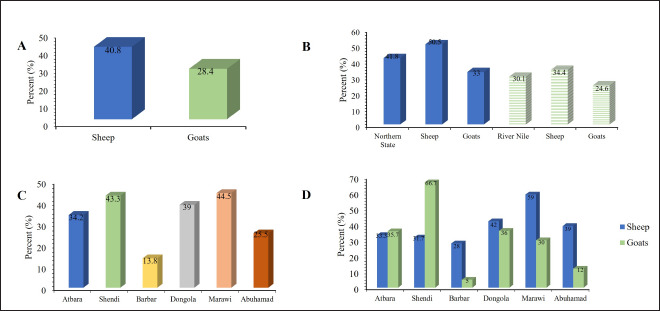
Seroprevalence of PPR antibodies among sheep, goats as detected by c-ELISA in Northern states of Sudan, according to species (A), Northern state (Solid color) and River Nile (Striated) (B), District (C) and species per District (D).

Antibodies against PPRV were found in 206 sheep and 125 goats. The overall detected seropositivity reached 35% (40.8 in sheep, 28.4 in goats). Table 1 and Figure 1 illustrate the details of the reactive sera according to species, state, and district.

### The Chi-square test

The statistical analysis revealed a significant correlation between PPR with species (*p* = 0.000), state (*p* = 0.000), and district (*p* = 0.000). State-wise Correlation between PPR and species showed a significant correlation in the Northern state (*p* = 0.000); nonetheless, no association between the PPR and species in River Nile (*p* = 0.013) was noticed (Table 2).

**Table 2.** Pearson Chi-square analysis of PPR according to species, state and district as detected by c-ELISA.

	Species	State	District	Northern State	River Nile state
NO	440	440	440	240	200
Pearson *Chi*-square	15.843^a^	13.777^a^	32.848^a^	12.593^c^	6.186^d^
df	1	1	5	1	1
Asymptotic Significance	0.000	0.000	0.000	0.000	0.013

### Determination of PPR antigen

By using IcELISA, PPR antigen was found in 7.7% of tested tissues; in River Nile State, it was 5% in sheep; in Northern State, 15.4% of sheep and 20% of goat lung tissue samples tested positive (Table 3).

**Table 3.** Detection of PPR antigen in sheep and goat lung tissue using IcELISA in Northern Sudan.

Location	Sheep lung tissue			Goat lung tissue			Total +Ve
	No. tested	+ve	%	No. tested	+ve	%	
River Nile State							
Atbara	50	3	6	–	–	–	6
Shendi	10	–	–	–	–	–	–
Barbar	–	–	–	–	–	–	–
Abuhamad	–	–	–	–	–	–	–
Northern State							
Dongola	7	1	14.3	3	1	33.3	20
Marawi	6	1	16.7	2	–	–	12.5
Total	73	5	6.8	5	1	20	7.7

### Detection of PPR genome

**Figure 2.**
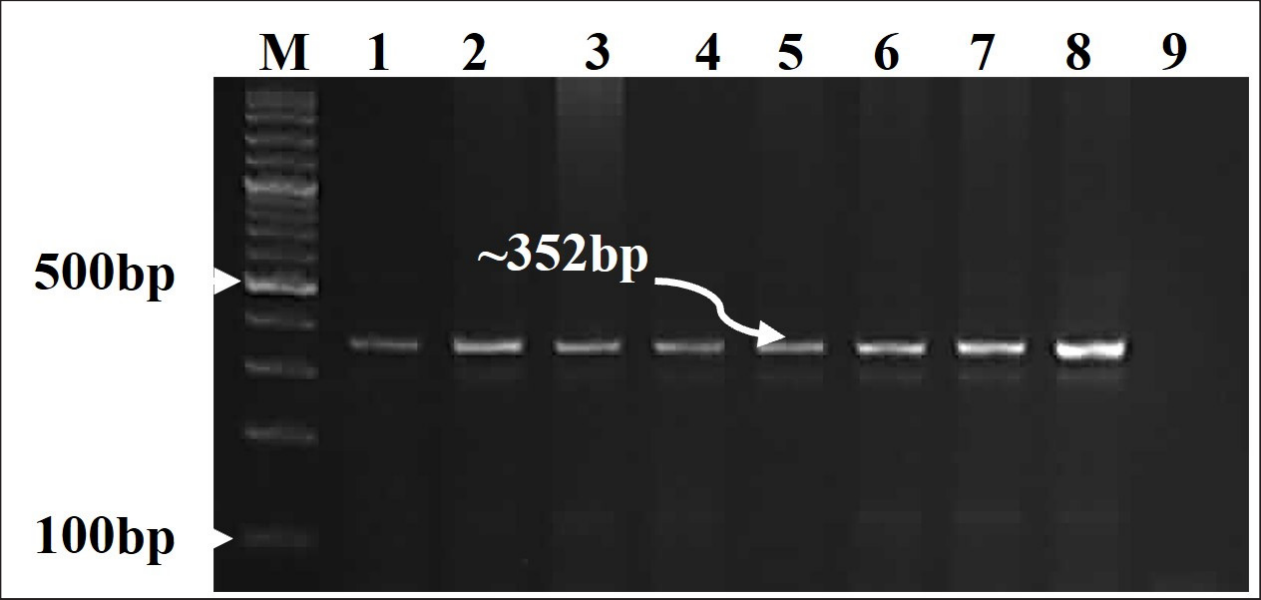
Ethidium bromide-stained agarose gel (1.5%). RT-PCR was carried out on RNA samples purified from lung tissues of infected sheep and goats using NP3 and NP4 primers. Lane M: 100 bp ladder, lane 1 and lane 8 control positive, lane 2–4 sheep samples, lane 5–7 goat sample, line 9 control negative.

All 6 RNA samples gave positive results for the *nucleoprotein gene*. Strong bands were visualized in the ethidium bromide-stained gel that corresponds exactly to the expected RNA size of the control positive nucleic acid (~352 bp) (Fig. 2).

## Discussion

PPRs are widely distributed, especially in Africa and Asia. In Sudan, variable prevalence rates were reported in different localities [20,25,27,31,32]. The present study focused on Northern Sudan, an area that was considered a disease-free zone and of the lowest reported prevalence, where the highest mortality rate was recorded during the first report of the disease in the area in 2002 [33]. Our results revealed an overall PPR seroprevalence of 40.8% in sheep and 28.4% in goats in Northern Sudan. Detected seroprevalence in each state was 34.4% in sheep, 24.6% in goats in the River Nile, 50.5% in sheep, and 33% in goats in the Northern State. This indicates more spread of the disease in the Northern State than River Nile State. Some more work in the River Nile State has been published, while work in Northern State is scarce. Concerning River Nile State, our results are in agreement with that detected (29% and 33% in sheep and 31% in goats) in the first report for PPR seroprevalence in River Nile State of Northern Sudan [22]. Slightly lower seroprevalence in sheep (26%) while a higher one in goats (40%) was detected [24]. Higher seroprevalence (46%) in sheep was reported in 2008 [25].

From that time onwards, PPR looks to be of more spread in the State; during 2008–2012, 63% of sheep and 33% of goat sera were tested positive in River Nile and Northern States [26]. The highest PPR seroprevalence (89% of sheep and 100% of goats) has been reported during 2016–2017 in River Nile State [27]. This obvious increase could be attributed to the increase in animal population in the state, besides the existence of one of the main animal markets in River Nile State and the easy movement of animals from areas of widely spread PPR in Central, Eastern, and Khartoum States. The picture in Northern State observed in this study showed that PPR seroprevalence was 50.5% in sheep and 33% in goats. This is obviously higher than the previously reported figures, 26% of sheep and 19% of goat sera [23], also recently detected (32%) during 2022 [28].

Generally, the spread of PPR in Northern Sudan is lower than in other parts of Sudan; this is mainly due to, firstly, the low animal population and the nature of animal management, as the main activity in Northern Sudan is agriculture. Animals are kept in small flocks either within the farming areas or near residential areas, so animals rarely come in contact with pasture and water as seen in other parts of Sudan. Northern Sudan is bordered by Egypt to the North, Western Sudan (Kordofan, Darfur) to the West, Eastern Sudan (Kassala) to the East, and Khartoum to the South. The prevalence of PPR in Northern Sudan is the lowest compared to other parts of Sudan, especially Western Sudan (Kordofan and Darfur), which is the richest area with an animal population where animals are kept in large flocks and are usually sharing natural pasture and water resources. Although both states are bordering Northern State, there is no direct access between them; this acts as a barrier against the spread of the disease.

In Kordofan, 75% seroprevalence in sheep was detected [20], 72.5% in sheep, 38% in goats [34], 41% [21], 75% in sheep [35], 61% in sheep, 64% in goats [31], 58% in sheep, 39% in goats [26], 58% in sheep and goats [28], and recently the highest seroprevalence, 100% in sheep and 97% in goats, was reported [36]. In Darfur, reported PPR seroprevalence was 49% in both species [21], 53% in sheep, 35% in goats [22], 68% in sheep [26], and 54% in sheep and goats [28]. The existence of PPR in countries bordering Western Sudan was reported in Chad [37,38] and in Libya, with 44% in sheep and 59% in goats [39]. To the North, PPR was reported in Egypt, 59% of goats and 57% of sheep [7], and 43% in goat sera were seropositive [40]. The second most animal-populated area is Central Sudan (Al Gezira and White Nile States). PPR seroprevalence in this area was 73% in sheep, 58% in goats [32], 62% in sheep, 54% in goats, 54% in sheep, 53% in goats in White Nile State [33,41], 70% in sheep, 67 % in goats [26], 90% in sheep, 81% in goats in Al AlGezira [21], 79% in sheep, 77% in goats at White Nile State [36], and 70% in sheep and goats at Blue Nile State [28].

The situation of PPR seroprevalence in Eastern Sudan States (Kassala, Gedarif, Port-Sudan), the third most animal-populated areas, is also higher than that detected in Northern Sudan. In Eastern Sudan, 91% seroprevalence in sheep was detected [32], 66% in sheep in Kassala [34], 39% in sheep, 60% in goats in Gedarif [24], 68% in sheep, and 57% in goats [26]. In countries bordering Eastern Sudan, the situation of PPR is similar; in Eritrea, PPR was confirmed by the detection of the viral genome [42], and serologically, 38% in sheep and 69% in goats in Ethiopia [3].

Within the two Northern States, the detected seroprevalence in the study was variable; in River Nile, there was no marked difference between localities, while for goats, the highest figure (67%) was observed in Shendi at the middle to the south of the state. In Northern State, the highest figure in sheep (59%) was reported in Marawi Province, which is far higher than the previously reported figure (26%) in the same province [23]. The highest figures for goats were in Dongola (36%) and then Marawi (30%), which is also far higher than the reported one (19%) in the same province [23]. The detected PPR seroprevalence in this study is considered low compared to other states of Sudan; nevertheless, the figures are alarming and indicate the increased spread of the disease in Northern Sudan.

In the present study, PPRV antigen was found in 5% of tested sheep lungs in the River Nile, while no goat sample was tested. In the Northern State, PPRV antigen was found in 15.4% of sheep and 20% of goat samples. The results are lower than the detected figures during 2008 in sheep in the River Nile and Eastern States (100%); it is expected as all samples in the previous study were collected during the PPR outbreak, and only 2 samples from Eastern and one sample from the River Nile State were tested [31]. However, the detected PPR antigen in all other parts of Sudan in the same study was higher than our results, where it was 38% in Western and 22% in Khartoum and Central States [32]. In White Nile State at Central Sudan, PPRV antigen was found in 7% of sheep lungs [43]; a higher figure was detected also in the same state [33]. In a more recent study, PPR antigen detected in sheep in the River Nile was similar to our results (6%); meanwhile, in goats, it was far higher (46%); the highest figures (67% in goats, 64% in sheep) were detected in Eastern State and (57% in sheep) in Kordofan at Western Sudan [26]. The highest PPR antigen prevalence (100% in goats, 84% in sheep) was reported during 2019 in Al AlGezira at Central Sudan [44] and 73% in sheep in Kassala at Eastern Sudan [45].

In this study, the PPR genome was detected in all ELISA-positive samples. The results, besides the confirmation of PPR’s existence in Northern Sudan, supported the results of the IcELISA, which is the main test applied for PPR diagnosis in Sudan; the same result was concluded previously [26,32].

## Conclusion

From this study, it was concluded that PPR, which is widely distributed in Sudan, has recently been extensively spread in Northern Sudan, which was considered a disease-free zone. More in-depth studies need to be done in more places in Northern Sudan to find out how the disease is spreading right now so that the right control measures can be put in place.

## List of abbreviations

bp, Base pair

cELISA, Competitive ELISA

IcELISA, Immunocapture ELISA

PPR, Peste des Petits ruminants

PPRV, PPR virus

RNA, ribonucleic acid

RT/PCR, reverse transcription PCR.
